# Indoor Fungal Contamination in Temporary Housing after the East Japan Great Earthquake Disaster

**DOI:** 10.3390/ijerph18063296

**Published:** 2021-03-23

**Authors:** Maiko Watanabe, Rumi Konuma, Naoki Kobayashi, Akiko Yamazaki, Yoichi Kamata, Kenichi Hasegawa, Noritaka Kimura, Naomi Tsurikisawa, Chiyako Oshikata, Yoshiko Sugita-Konishi, Kosuke Takatori, Hiroshi Yoshino, Yukiko Hara-Kudo

**Affiliations:** 1 Division of Microbiology, National Institute of Health Sciences, Kawasaki-ku, Kawasaki, Kanagawa 210-9501, Japan; ykudo@nihs.go.jp; 2 Tokyo Metropolitan Industrial Technology Research Institute, Koto-ku, Tokyo 135-0064, Japan; konuma.rumi@iri-tokyo.jp; 3 Department of Food and Life Science, School of Life and Environmental Science, Azabu University, Chuo-ku, Sagamihara, Kanagawa 252-5201, Japan; n-kobayashi@azabu-u.ac.jp (N.K.); yoshikoni2020@gmail.com (Y.S.-K.); 4 Department of Veterinary Medicine, Faculty of Agriculture, Iwate University, Morioka, Iwate 020-8550, Japan; ayamazak@iwate-u.ac.jp (A.Y.); y-kamata@cs.kinran.ac.jp (Y.K.); 5 Department of Food and Nutrition, Faculty of Human Life Science, Senri Kinran University, Suita, Osaka 565-0873, Japan; 6 Department of Architecture and Environment Systems, Faculty of Systems Science and Technology, Akita Prefectural University, Yurihonjo, Akita 015-0055, Japan; haseken@akita-pu.ac.jp; 7 Department of Bioengineering, Nagaoka University of Technology, Nagaoka, Niigata 940-2188, Japan; nkimura@vos.nagaokaut.ac.jp; 8 Department of Allergy and Respirology, Hiratsuka City Hospital, Hiratsuka, Kanagawa 254-0065, Japan; n-tsuri@hiratsuka-city-hospital.com (N.T.); c-oshi@hiratsuka-city-hospital.com (C.O.); 9 Department of Pulmonology, Yokohama City University Graduate School of Medicine, Yokohama, Kanagawa 236-0004, Japan; 10 Center for Fungal Consultation Japan, Oota-ku, Tokyo 145-0067, Japan; takatori@kabisoudan.com; 11 Tohoku University, Aoba-ku, Sendai, Miyagi 980-0862, Japan; yoshino@sabine.pln.archi.tohoku.ac.jp

**Keywords:** mycoflora, Great East Japan Earthquake, temporary housing, allergic fungi, *Aspergillus*

## Abstract

To understand fungal contamination in the indoor environment of the disaster region, a field survey was performed to measure the number of fungal counts and identify isolates in the indoor air of prefabricated temporary housing, privately independent-housing, and rented apartments flooded by the East Japan Great Earthquake disaster tsunami. As a result, the period with the highest detected fungal count was from the rainy season to summer in independent-housing and rented apartments. Moreover, in the temporary housing, the fungal number increased further in winter as indicated by the maximum fungal-number throughout the measurement period. The detection frequency of *Aspergillus* species was relatively higher in the indoor air of temporary housing than in typical housing in the non-disaster area. Since *Aspergillus* is known as an allergenic genus, it requires careful attention to the health risk for residents. The extremely high level of fungal condensation in indoor air possibly occurred due to high relative humidity and loss of heat insulation in the building attics. It is suggested that this problem commonly happened in the cold region including the entire disaster region of the East Japan Great Earthquake.

## 1. Introduction

In general, fungal spores and other dispersed fungal structures normally float in the indoor air environment, and fungi are a ubiquitous taxon both in natural and man-made environments. However, when an indoor environment has suitable conditions for fungal growth, overgrowth can be specifically observed in rooms [1].

When the Great East Japan Earthquake happened in March 2011, approximately 60,000 temporary houses were built in seven prefectures: Iwate, Miyagi, Fukushima, Ibaraki, Tochigi, Chiba, and Nagano [2]. Because indoor fungal occurrence has become a serious issue among the residents in temporary houses in 2011, the government and residents’ association received a number of complaints or requests for the implementation of countermeasures. In a large-scale disaster like this, people are compelled to live in the remaining collapsed and temporary houses for a long period of time. The indoor environment may be in poor conditions due to inappropriate temperature/humidity or insufficient cleaning, resulting in fungal overgrowth. In such indoor environments, the fungal community may lose species diversity, and it may display higher fungal counts than those in typical housing in non-disaster areas. Because residents are exposed by these dominant fungal species at high concentrations, the health risk needs to be examined. It is known that *Penicillium* and *Cladosporium* species can be frequently detected in almost every environment in Japan [3,4,5], whereas *Aspergillus* species sometimes grow easily in indoor environments in typical housing in non-disaster areas [1]. The human health hazards caused by fungal contamination of the indoor-air environment include infectious diseases [6,7,8], mycotoxicosis [9], and allergies [10,11,12]. The health risks of damages to a person vary according to the kind of the fungi. *Aspergillus* species are well known as a major pneumonic and allergenic fungus, and a representative species is *A. fumigatus* [13]. In particular, allergies, a group of disorder, occur in all age groups—from infants to the elderly—affecting a large number of patients with various symptoms. Thus, when evaluating human health hazards caused by fungi, it is important to make a proper evaluation by focusing on more hazardous allergic risks. This study aimed to examine and understand the health risk caused by the exposure of abnormal fungal community in temporary houses with a viewpoint from a number of fungi and detected fungal species.

## 2. Materials and Methods

**Figure 1:**
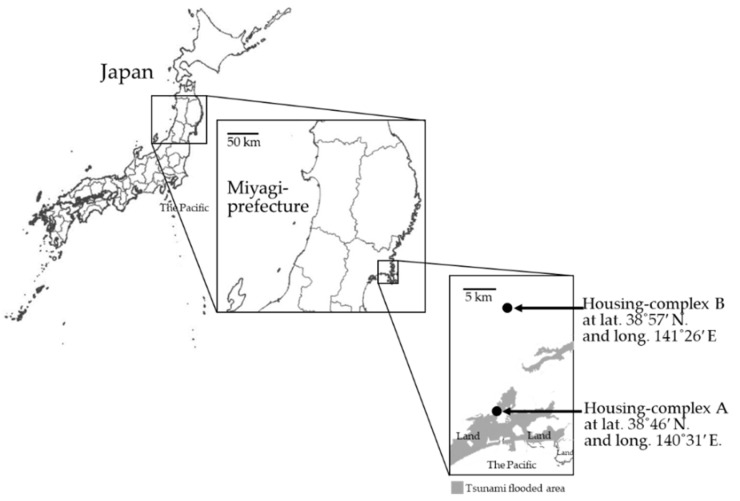
The map of the locations of two temporary housing-complexes tested in this study. This map is based on the GSI maps published by Geospatial Information Authority of Japan. The gray-colored area is tsunami flooded.

**Figure 2:**
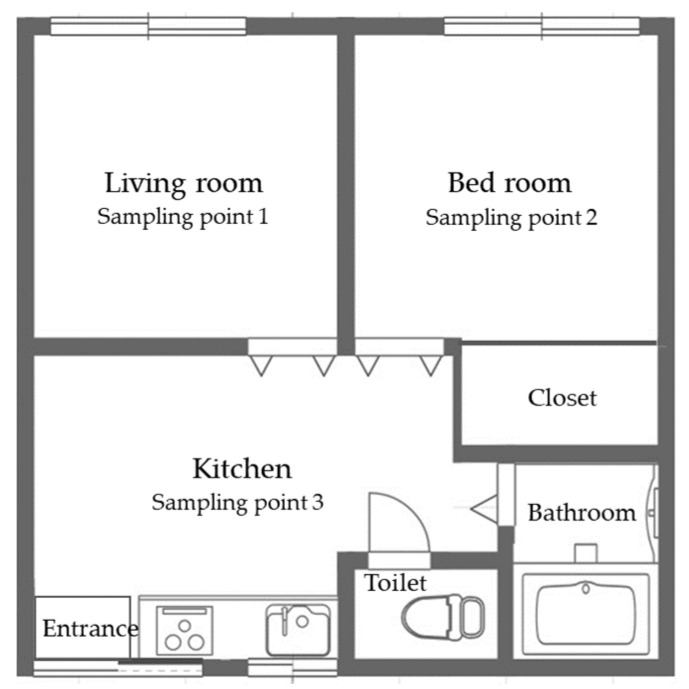
The floor plan of a typical temporary house and sampling points in rooms tested in this study.

We selected the temporary housing-complex A, an area near the tsunami-flooded district, and temporary housing-complex B, an area in a high elevation district far away from the flooded district (Figure 1). In addition, we also collected samples from other housing complexes within a distance of ten kilometers from the complexes A or B for comparison. Regarding the sampling period, we collected samples four times a year covering four seasons (rainy season, summer, autumn and winter) allowing us to comprehensively understand the annual fungal contamination conditions. We collected samples from seven houses in July, 14 houses in August, 12 in October in 2012, 19 houses in March in 2013, for a total of 52 homes. The 16 homes sampled in this study belonged to the temporary housing-complex A, and 22 homes sampled in this study belonged to the temporary housing-complex B, respectively. Furthermore, we conducted an additional study to measure the relative ratio of *Aspergillus* counts in the fungal community. We collected samples in 17 temporary houses in temporary housing-complexes A and B from October 2014 to March 2015. Air samples were collected from three points located in the living room, bedroom, and kitchen in each house as well as from the outside air within the same temporary housing-complex sites. The floor plans of the houses tested in this study are described in Figure 2. We collected samples of the air in each room and outdoor air on five dichloran rose-bengal chloramphenicol plates (DRBC; Thermo Fisher Scientific Inc., Waltham, MA, USA) and dichloran-glycerol 18 (DG18; Thermo Fisher Scientific Inc.) with an air sampler (Air Ideal 3P; Biomerieux Inc., Marcy l’Etoile, France). Ten or 100 L-air was collected on an agar plate. After an incubation period of seven days, fungal colonies on the agar plates were coun8ted, and counts per one liter of air were calculated. The counts were converted into colony-forming-unit per 1 m^3^ air (CFU/m^3^) with the table attached to the air-sampler based on the most probable number method. The detection upper-limits were 32,640 CFU/m^3^ in the method used in July, and 163,200 CFU/m^3^ in the method used in August and March, respectively. We evaluated the level of fungal contamination in each room in comparison with the border count of 1000 CFU/m^3^ recommended by Architectural Institute of Japan [14]. We also collected samples in flooded rental apartment and privately-independent housing located in nearby areas with temporary-housing in Miyagi-prefecture for comparison with fungal counts in temporary-housing.

In each house, the indoor and outdoor temperature and relative humidity were monitored in the living and bedrooms every 15 or 30 min from July in 2012 to March in 2013. They were measured using thermohydrometers (Ondotori TR-72Ui, T&D Corporation, Nagano, Japan) placed 100–120 cm above the floor.

All colonies on DRBC and DG-18 agar plates were subclassified into genus-based groups by the stereoscopic and microscopic features as the genus *Aspergillus, Cladosporium* and *Penicillium*, yeasts, and others [15,16]. Then, the colonies belonging to the dominant species in each room was isolated on a new potato dextrose agar plate (PDA; Eiken Inc., Tokyo, Japan) to identify the species level.

For the statistical analysis of the data in this study, the Student’s *t*-test was performed for a comparison of fungal counts, temperature, and humidity using the BellCurve for Excel software (Social Survey Research Information Co., Ltd., Tokyo, Japan).

## 3. Results and Discussion

**Figure 3:**
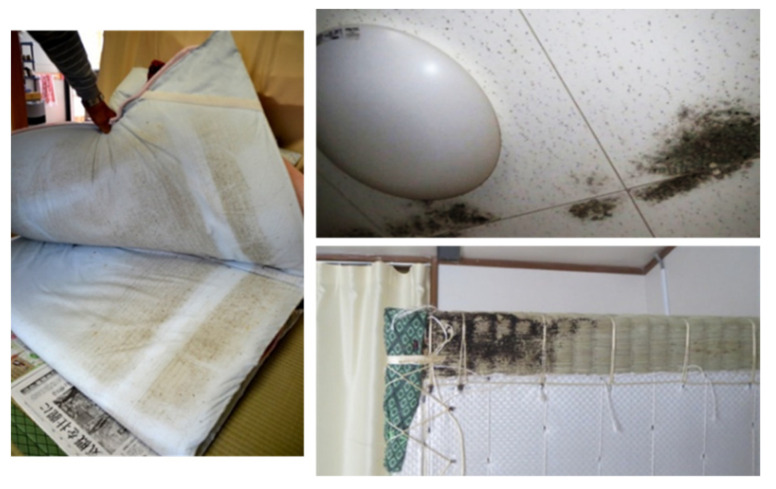
Fungal contamination in temporary housings.

We conducted a fungal survey in 52 prefabricated temporary houses. After checking the fungal growth condition in the rooms, we found a number of fittings and furniture with fungal overgrowth in both housing complexes (Figure 3).

**Figure 4:**
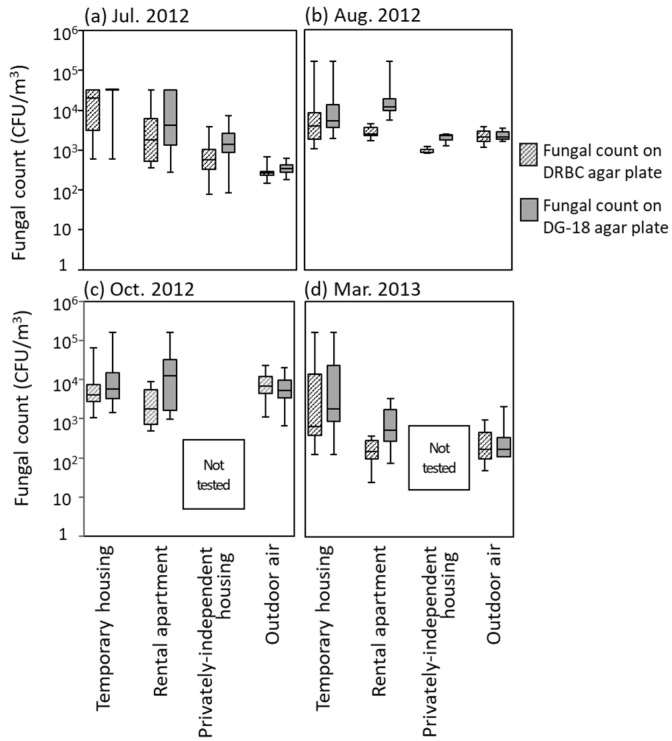
Comparison of fungal counts in various types of housing. We collected samples four times a year in 52 homes for covering four seasons; (**a**) rainy season in July 2012, (**b**) summer in August 2012, (**c**) autumn in October 2012, and (**d**) winter in March 2012. Boxplots were formed in each data-set of fungal counts on DRBC agar plates or them on DG-18 agar plates.

We performed a comparative analysis among the housing types based on fungal contamination level per m^3^ of indoor air in each temporary housing, flooded rental apartments, and privately independent housing, presented as a boxplot (Figure 4). We made a comparison among these types of housing with hygrophilic fungi measured by DRBC and xerophilic fungi measured by DG-18. However, the differences between the fungal counts observed in Figure 4 were not statistically significant. The results indicate that the median fungal count on DG-18 was higher than on DRBC for each housing type in all seasons. It is suggested that the count of xerophilic fungi measured by DG-18 was higher than that of hygrophilic fungi measured by DRBC; therefore, DB-18 is more suitable for fungal count investigation in indoor environments.

The recommended level of fungal counts in typical buildings in a non-disaster area is less than 1000 CFU/m^3^ mentioned above. A recorded fungal count much higher than this level (63,200 CFU, the upper limit of detection in this study) was detected in the three types of housing tested in this study in all four seasons (Figure 4). It was confirmed that fungal counts in temporary housing remained at a much higher level than 1000 CFU/m^3^ throughout the year, including the winter season. On the other hand, the maximum fungal count/m^3^ in tsunami-flooded housing in July 2012 was 7440 CFU in privately independent housing and 32,640 CFU in rental apartments; the one in August 2012 was 2420 CFU in privately independent housing and 163,200 CFU in rental apartments (Figure 4). This result indicated that the fungal contamination level in rental apartments was higher than in privately independent housing during the spring and rainy seasons. The high fungal counts in rental apartments were maintained during autumn, and then decreased to a maximum of 3425 CFU/m^3^ in winter. This confirmed that indoor fungal contamination could not be observed in tsunami-flooded housing more than one year after the disaster. We need to understand the hygienic conditions of prefabricated temporary housing in which fungi easily grow.

**Figure 5:**
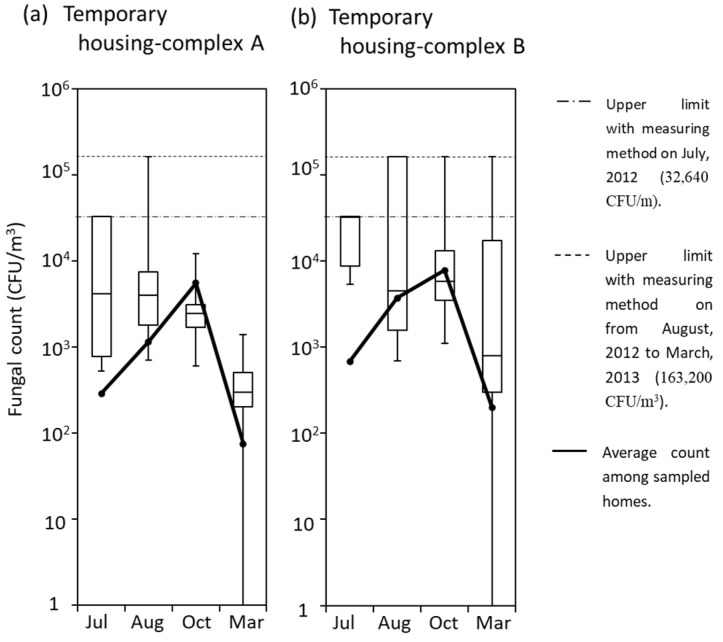
Comparison among fungal counts at four seasons in two temporary housing-complexes; (**a**) temporary housing-complex A and (**b**) temporary housing-complex A. We collected samples four times a year for covering four seasons (rainy season in July, summer in August, autumn in October, and winter in March). The 16 homes in the temporary housing-complex A and the 22 homes in the complex B were sampled. Boxplots were formed in each data-set of fungal counts on DG-18 agar plates.

A comparison of fungal counts in each month between the two temporary housing complexes is shown in a boxplot (Figure 5). Because the results of hygrophilic and xerophilic fungi indicated the same tendency (data not shown), only the xerophilic fungal counts determined by DG-18 were described. The differences between the fungal counts observed in Figure 5 were not statistically significant. However, in temporary housing-complex B in October and March, there was a noticeable wide variation in fungal counts, with a highest fungal count of 163,200 CFU/m^3^ as an upper limit of detection in several housings. These features were not confirmed in housing-complex A.

**Figure 6:**
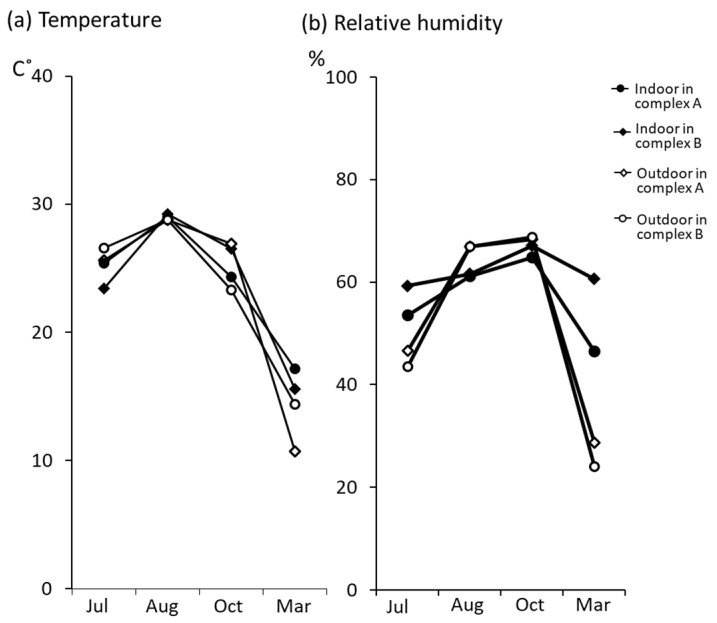
Time drift of temperature and relative humidity in two temporary housing-complexes. We measured temperature and relative humidity 4 times a year for covering four seasons (rainy season in July, summer in August, autumn in October, and winter in March). (**a**) Temperature and (**b**) relative humidity. The 16 rooms in the temporary housing-complex A and the 22 rooms in the complex B were sampled, and the average were calculated in rooms in each complex.

In temporary housing-complexes A and B, we measured the temperature and relative humidity; then, a comparative analysis was conducted in different seasons and the temporary housing-complexes (Figure 6). The results showed that the variation in indoor temperature was almost consistent with the outdoor temperature in both temporary housing complexes. We also found that the average indoor humidity in temporary housing-complex B was significantly higher than in complex A in March (*p* > 0.05), in spite of almost the same outdoor humidity. Both complexes showed similar behavior regarding the drift of temperature. It was revealed that high relative humidity was one of the large differences in environmental factors between the two complexes.

We discuss the factors why housing with extremely high fungal count was occasionally found in the temporary housing-complexes tested in this study, especially in complex B during winter. Several studies have reported that high relative humidity enhances fungal growth [1,17]. Figure 6 shows that the main environmental factor which distinguishes the housing in complex B is high humidity in winter. Shinohara et al. [18] reported that temporary housings built in Fukushima-prefecture after the Great East Japan Earthquake had higher levels of indoor airborne fungal counts (around 4000 CFU/m^3^) than those in typical housing at a non-disaster area, correlating with the proportion of time in a period, when relative humidity was greater than 70%; this was supported by the results of this study. Therefore, it was suggested that the high relative humidity in winter caused the high fungal counts in housing-complex B. On the other hand, in temporary housing built at the time of the Chuetsu Offshore Earthquake in Niigata prefecture, fungal overgrowth was found on the surface of some indoor building materials due to dew condensation during winter. A large amount of dew condensation on the windows and walls was observed in many temporary housing complexes in Miyagi prefecture, including temporary housing-complex B in winter (personal observation). The result indicated that in the temporary housing tested in this study, except for housing-complex B, the highest fungal count of 2700 CFU/m^3^ recorded in March was relatively not high. Only the housing-complex B had an extremely high fungal count (163,200 CFU/m^3^ in Figure 5), possibly because only these visible dew condensations are not a strong cause of high fungal counts. In addition, in temporary housing-complex B, fungal overgrowth was frequently visually confirmed on ceiling panels (Figure 3). Watanabe et al. previously reported that the moldy part of the ceiling panel was completely matched with a locally lowered temperature on the ceiling panel surface in several fabricated temporary houses [19]. One of the factors for high humidity and locally lowered temperature on the ceiling panel surface could be a problem in terms of the building materials used and the loss of heat insulation. Decorative wooden board and decorative gypsum board were used as ceiling panels in temporary housing-complex A and temporary housing-complex B, respectively. Furthermore, the heat insulation could be another factor. In a previous study, Watanabe et al. checked the heat insulation condition in the attic of houses by removing the moldy ceiling panels in prefabricated temporary housings. They found a partial loss of heat insulation, such as partially insufficient glass wool or damaged insulation sheet in all of the checked places [19]. The material should be originally attached on a ceiling without a gap for attic insulation. Since the abovementioned ceiling panels’ temperature was lower in winter, it was assumed that the water content in decorative gypsum board could be increased locally. Because gypsum boards have superior moisture absorption, it is thought that gypsum boards have the ability to adjust the moisture in typical housing in non-disaster areas. However, as water content increases under the high humidity environment, it is thought that it is then in the most suitable condition for fungal growth [17,20]. This condition might be considered as a factor for high fungal counts observed indoors. It was thought that the phenomenon observed in the temporary housing-complex B represents a change that happened in the dwelling dampness. Thus, the use of these building materials to construct temporary housing in a cold region requires careful consideration. However, because highly concentrated fungal contamination of indoor air in temporary housing also took place in multiple temporary housing complexes besides temporary housing-complex B, it was unlikely that only the ceiling panel materials and high indoor humidity could have caused the contamination. In the future, it will be necessary to consider and examine various conditions, such as sunlight, underfloor condition, and residents’ way of living.

**Figure 7:**
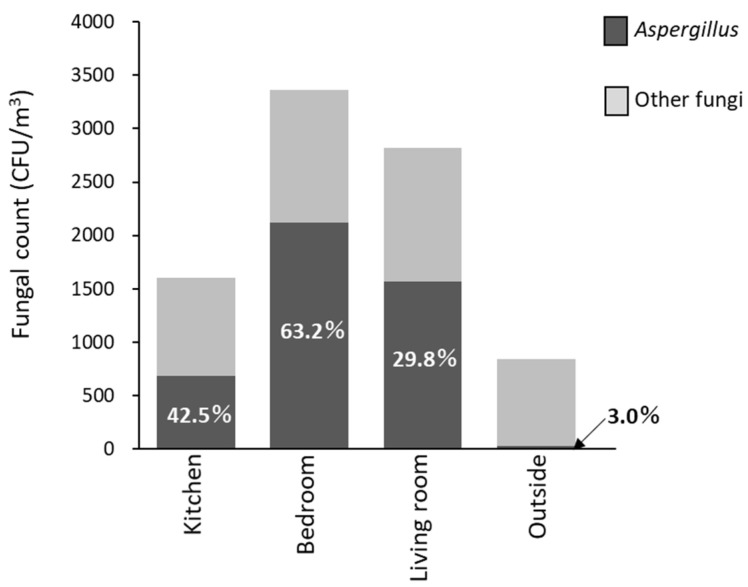
Ratio of *Aspergillus* in fungal count in the indoor-air of temporary houses during the autumn and winter months. We collected samples in 17 temporary houses from October 2014 to March 2015. Bar-plots represent fungal counts on DG-18 agar plates.

Furthermore, we additionally collected samples in 17 temporary houses from October 2014 to March 2015 with attention to the season from autumn to winter when humidity increased in the temporary housing (Figure 6), and then, we made a comparison of the proportion of *Aspergillus* species (including *Eurotium* species as a teleomorph of *Aspergillus* species) in each room (Figure 7). The results showed that *Aspergillus* species was detected in the kitchen (42.5%), bedroom (63.2%), and living room (29.8%), while only 3.0% was detected in outdoor air. It was reported that *Aspergillus* species can be detected at levels of 18.1–24.1% in typical housing in a non-disaster area [4,5,21]. The detection frequency of *Aspergillus* species was relatively higher in the indoor air of temporary housing than in typical housing in a non-disaster area. Several researchers have previously reported that *Aspergillus versicolor*, *Aspergillus* section *Restricti* and *Eurotium* species have good growth in high relative humidity and relatively cold temperature in damp buildings [1,13,22]. Several clinical case studies of fatal pneumonia and allergic bronchopulmonary mycosis by these species of *A. versicolor*, *Aspergillus* section *Restricti* and *Eurotium* were previously reported [12,13]. In our study, *A. fumigatus* was almost not detected, however, species other than *A. fumigatus* were often detected (data not shown). To evaluate the allergic risk in a temporary housing room, the allergenicity of these fungal species needs to be sufficiently considered.

It can be easily predicted that a number of disasters, such as earthquake and floods, will take place in Japan in the future. To plan for preventive measures against fungal contamination in temporary housing constructed at the time of disasters and ensure a high quality of life of residents, data collection/analysis should be done to investigate the factors causing fungal contamination in temporary housing.

## Data Availability

The data presented in this study are available on request from the corresponding author. The data are not publicly available to protect confidentiality of the research subjects.
